# Supplementary material for: North Atlantic oscillation controls multidecadal changes in the North Tropical Atlantic−Pacific connection
Source: Nat Commun. 2023 Feb 16;14:862. doi: 10.1038/s41467-023-36564-3 (PMC9931689; doi:10.1038/s41467-023-36564-3)
Supplement: Supplementary file 1 — Supplementary information [file 41467_2023_36564_MOESM1_ESM.pdf]

Supplementary Materials for

North Atlantic Oscillation controls multidecadal changes in the North Tropical Atlantic–Pacific connection

This PDF file includes:

Supplementary Figure 1. North Tropical Atlantic (NTA)–El Niño-Southern Oscillation (ENSO) connection during different periods.

Supplementary Figure 2. Temporal evolution of the North Tropical Atlantic (NTA)–El Niño-Southern Oscillation (ENSO) connection.

Supplementary Figure 3. Temporal evolution of the North Tropical Atlantic (NTA)–El Niño-Southern Oscillation (ENSO) connection and Atlantic multidecadal oscillation (AMO).

Supplementary Figure 4. Composite differences of divergence, precipitation, and specific humidity.

Supplementary Figure 5. Regressions with respect to the spring North Tropical Atlantic (NTA) sea surface temperature (SST) in the pre-industrial model simulations of the Community Earth System Model (CESM).

Supplementary Figure 6. Enhanced equatorial Atlantic precipitation response and North Tropical Atlantic (NTA) sea surface temperature (SST) persistence in the positive-North Atlantic Oscillation (NAO) forcing experiments.

Supplementary Figure 7. Relationship between the North Atlantic Oscillation (NAO) and North Tropical Atlantic (NTA) mixed layer depth (MLD).

Supplementary Figure 8. Temporal evolution of the North Tropical Atlantic (NTA)–El Niño-Southern Oscillation (ENSO) connection, the North Atlantic Oscillation (NAO) index, and the NTA sea surface temperature (SST) persistence.

Supplementary Figure 9. Imposed North Tropical Atlantic (NTA) sea surface temperature (SST) forcings in the coupled general circulation model (CGCM)

experiments.

Supplementary Figure 10. Atlantic multidecadal oscillation (AMO)-based linear model for predicting multidecadal North Atlantic Oscillation (NAO) index.

Supplementary Figure 11. Imposed North Atlantic Oscillation (NAO) forcing in coupled general circulation model (CGCM) experiments.

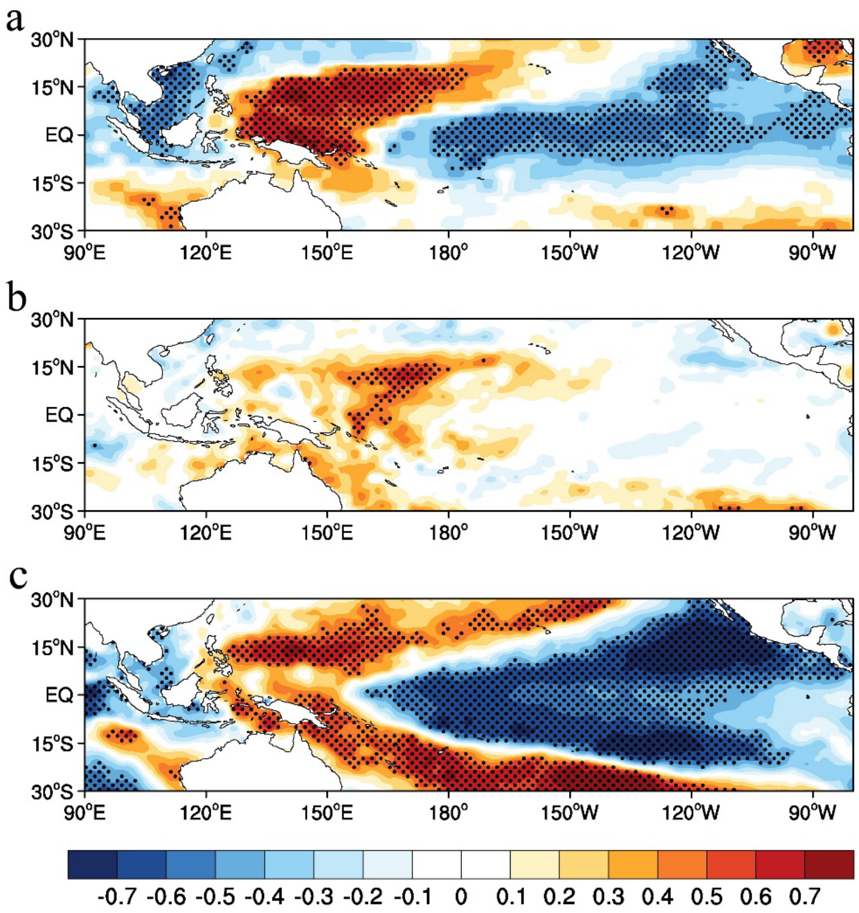

Supplementary Figure 1. North Tropical Atlantic (NTA)–El Niño-Southern Oscillation (ENSO) connection during different periods. Correlations between the boreal spring (MAM0) NTA sea surface temperature (SST) index and subsequent winter (D0JF1) tropical Pacific SST anomalies during 1913–1939 (P1) (**a**), 1947–1985 (P2) (**b**), and 1993–2010 (P3) (**c**). The impact of the previous winter (D–1JF0) ENSO has been removed from the MAM0 NTA SST index using linear regression with respect to the Niño3.4 index. Dots indicate the correlations significant at or above the 95% confidence level.

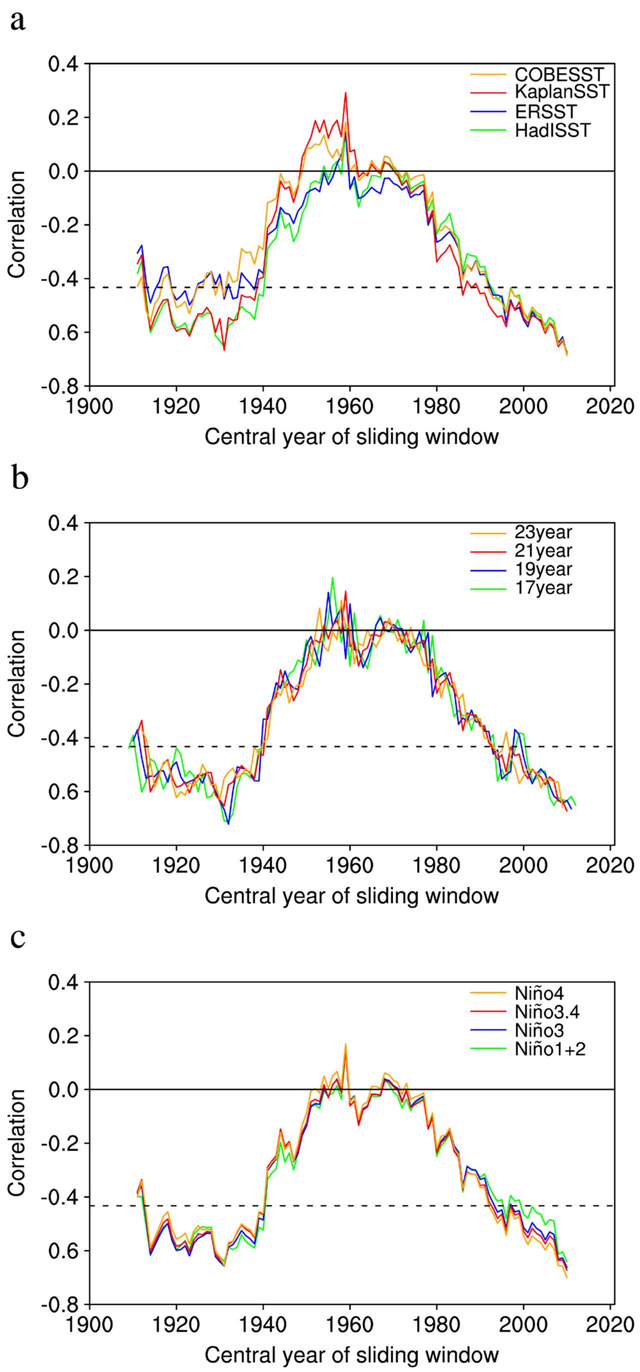

Supplementary Figure 2. Temporal evolution of the North Tropical Atlantic (NTA)–El Niño-Southern Oscillation (ENSO) connection. The 21-year sliding correlation coefficients between the boreal spring (MAM0) NTA sea surface temperature (SST) index and subsequent winter (D0JF1) Niño3.4 index during 1900–2021 based on different SST datasets (**a**), different lengths of the moving window (**b**), and different ENSO indices (**c**). The impact of the previous winter (D−1JF0) ENSO has been removed from the MAM0 NTA SST index using linear regression with respect to the Niño3.4 index, and the horizontal dashed line indicates the 95% confidence level.

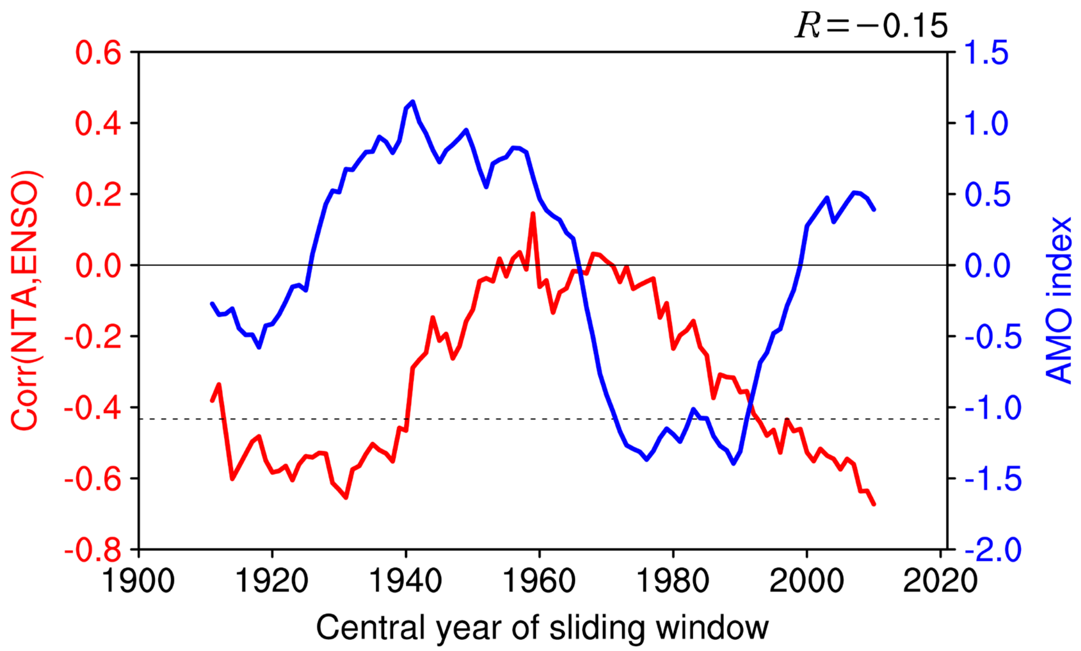

Supplementary Figure 3. Temporal evolution of the North Tropical Atlantic (NTA)–El Niño-Southern Oscillation (ENSO) connection and Atlantic multidecadal oscillation (AMO). The 21-year sliding correlation coefficients between the boreal spring (MAM0) NTA sea surface temperature (SST) index and subsequent boreal winter (D0JF1) Niño3.4 index (red line; left axis), and the 21-year running averages of the normalized AMO index during the previous boreal winter–spring seasons (D−1JFMAM0) (blue line; right axis) for the period 1900–2021. The horizontal dashed line indicates the 95% confidence level for the sliding correlation coefficients. The correlation coefficient between the NTA–ENSO connection and AMO is −0.15 (not significant even at the 90% confidence level).

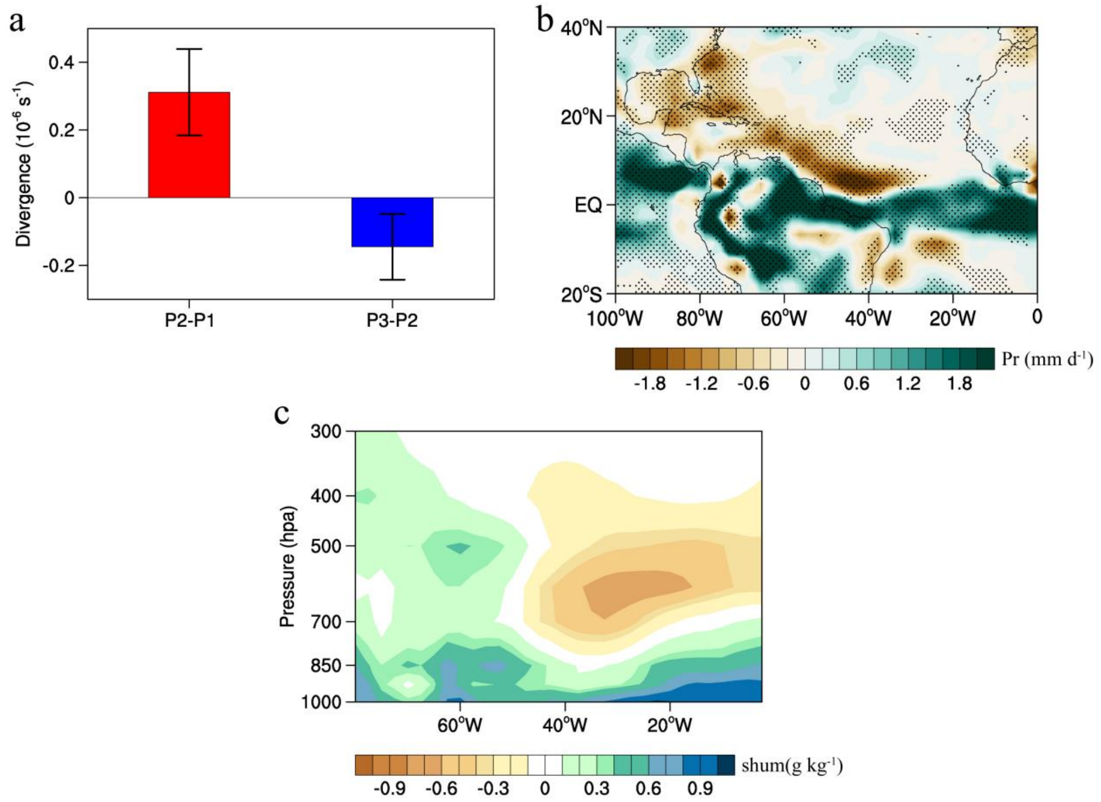

Supplementary Figure 4. Composite differences of divergence, precipitation, and specific humidity. (a) Composite differences of 850-hPa wind divergence (10^{-6} s^{-1}) during the previous boreal winter–spring seasons (D−1JFMAM0) averaged over the equatorial Atlantic (5°S to 5°N and 70°W to 0°) between 1947–1985 (P2) and 1913–1939 (P1) (red bar) and between 1993–2010 (P3) and 1947–1985 (P2) (blue bar). Error bars indicate the 95% confidence intervals. **(b)** Composite differences of tropical Atlantic spring (MAM0) precipitation anomalies (shading; mm day^{-1}) between the positive phase of the North Atlantic Oscillation (NAO) (1993–2010) and its negative phase (1948–1985). Dots indicate the precipitation anomalies significant at the 95% confidence level. (**c**) Composite differences of spring (MAM0) specific humidity (g kg^{-1}) averaged between 5°S−5°N at different levels between the positive phase of the NAO (1993–2010) and its negative phase (1948–1985). Because the NCEP/NCAR reanalysis precipitation and specific humidity data are available since 1948, we analyzed composite differences in precipitation and specific humidity anomalies between the periods 1993–2010 and 1948–1985.

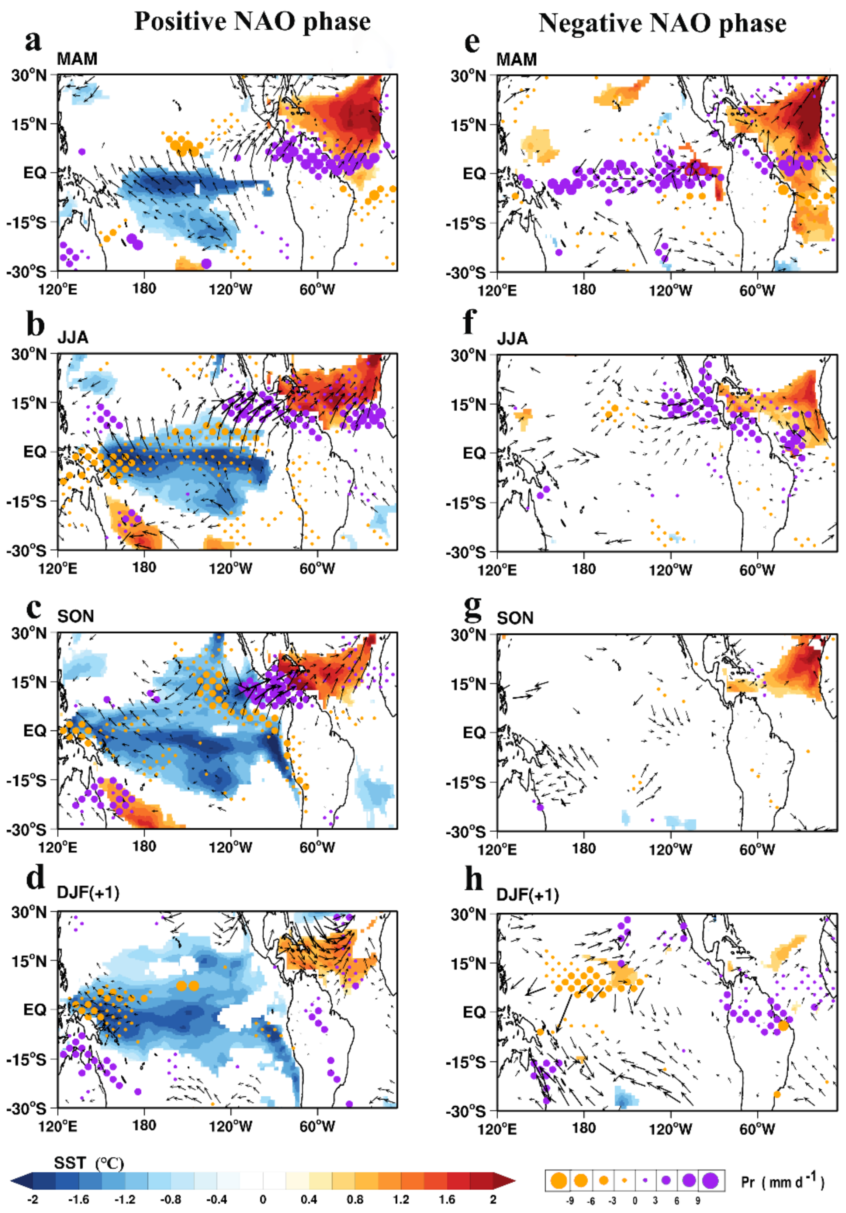

Supplementary Figure 5. Regressions with respect to the spring North Tropical Atlantic (NTA) sea surface temperature (SST) in the pre-industrial simulations of the Community Earth System Model (CESM). (**a–d**) Regressions of the 3-month averaged SST (°C; shading), 850-hPa winds (m s^{-1}; vectors), and precipitation (mm day^{-1}; stippled) anomalies with respect to the MAM0 NTA SST index during positive North Atlantic Oscillation (NAO) phases for MAM0 (a), JJA0 (b), SON0 (c) and D0JF1 (d) seasons in the pre-industrial simulations of the CESM. (**e–h**) As in (**a–d**), but during negative NAO phases for MAM0 (e), JJA0 (f), SON0 (g) and D0JF1 (h) seasons. The impact of the previous winter (D−1JF0) El Niño-Southern Oscillation (ENSO) has been removed from the MAM0 NTA SST index using linear regression with respect to the Niño3.4 index. Only SST, 850-hPa winds, and precipitation anomalies significant at or above the 95% confidence level are shown.

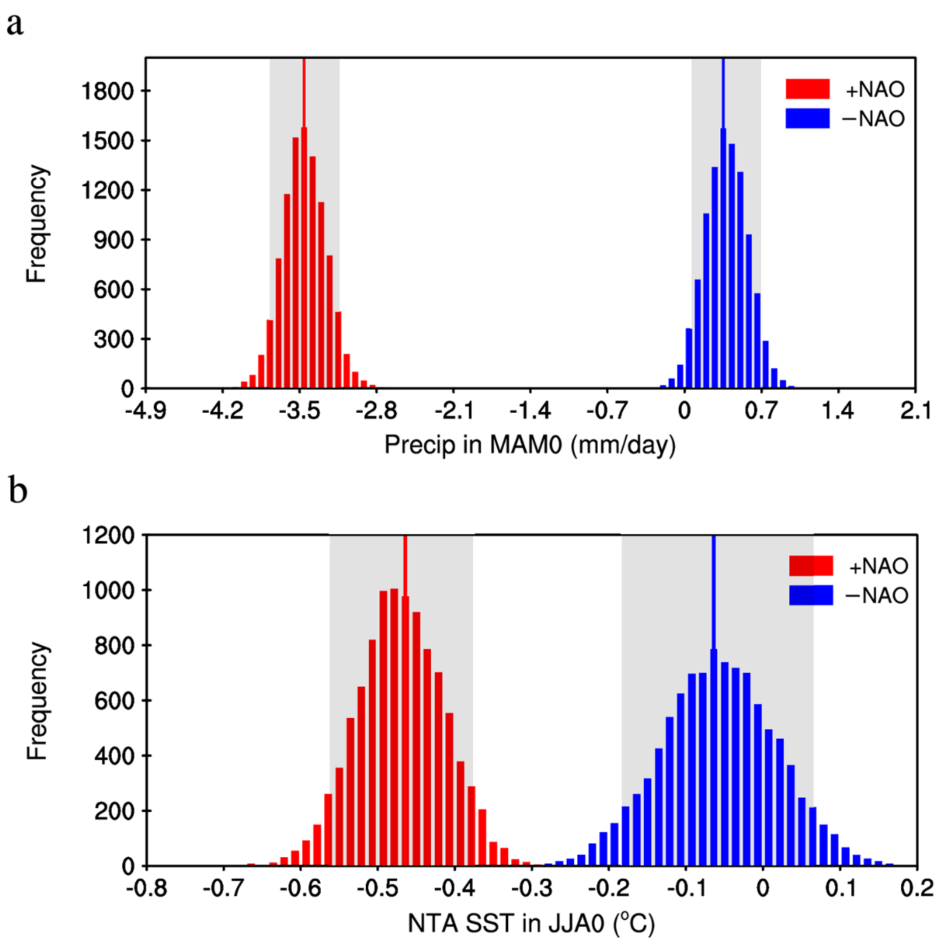

Supplementary Figure 6. Enhanced equatorial Atlantic precipitation response and North Tropical Atlantic (NTA) sea surface temperature (SST) persistence in the positive-North Atlantic Oscillation (NAO) forcing experiments. Histograms of 10,000 realizations of the bootstrap method for the boreal spring equatorial Atlantic precipitation response (**a**) and summer NTA SST anomalies (**b**) in the positive-NAO (red) and negative-NAO (blue) forcing experiments (see Methods). The equatorial Atlantic precipitation response is measured by precipitation anomalies area-averaged over the equatorial Atlantic (5°S to 5°N and 70°W to 0°) during boreal spring (MAM0), and SST anomalies are averaged over the NTA region during boreal summer (JJA0). The red and blue vertical lines indicate the mean values of 10,000 inter-realizations for the positive-NAO and negative-NAO forcing experiments, respectively. Gray shaded regions indicate the respective doubled SDs (the 95% confidence interval based on the normal distribution) of the 10,000 inter-realizations. Because the positive NAO forcing induces negative NTA SST anomalies, both spring precipitation anomalies in (**a**) and summer NTA SST anomalies in (**b**) under the positive NAO forcing are negative.

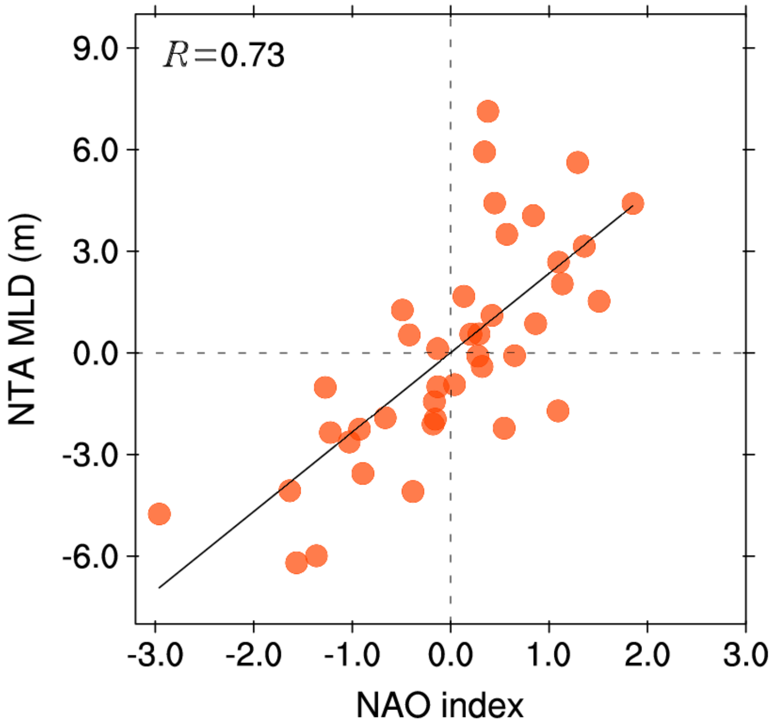

Supplementary Figure 7. Relationship between the North Atlantic Oscillation (NAO) and North Tropical Atlantic (NTA) mixed layer depth (MLD). Scatterplot of the winter–spring (D−1JFMAM0) NAO index versus the spring (MAM0) MLD area-averaged over the NTA region. The linear regression line is shown by the solid line, with the correlation coefficient (*R*) being 0.73 (significant at the 99.9% confidence level).

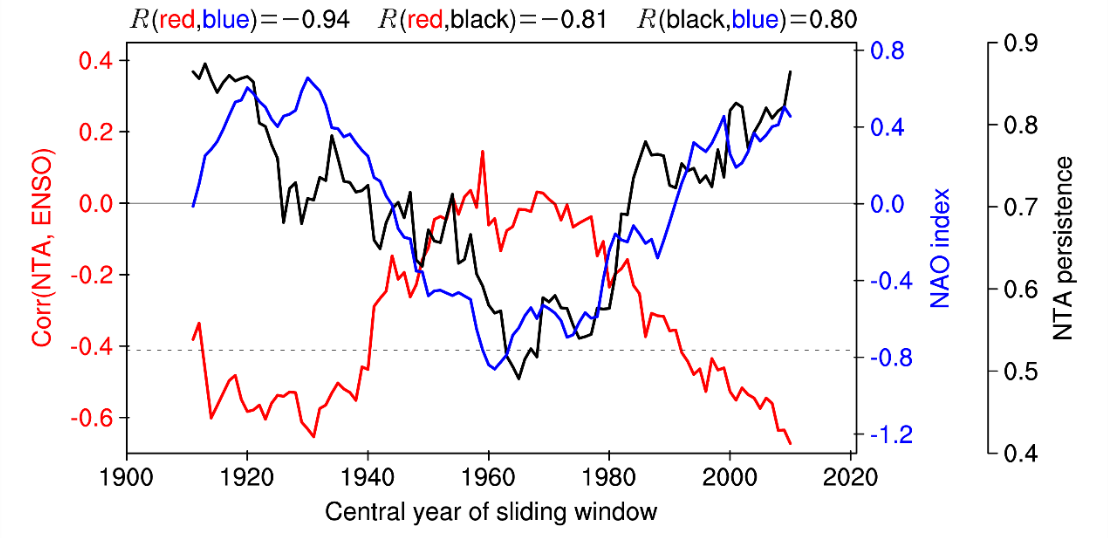

Supplementary Figure 8. Temporal evolutions of the North Tropical Atlantic (NTA)–El Niño-Southern Oscillation (ENSO) connection, the North Atlantic Oscillation (NAO) index, and the NTA sea surface temperature (SST) persistence. The red line denotes the 21-year sliding correlation coefficients between the boreal spring (MAM0) NTA SST index and subsequent boreal winter (D0JF1) Niño3.4 index, the blue line is the 21-year running averages of the normalized NAO index during the previous boreal winter–spring seasons (D–1JFMAM0), and the black line is the 21-year sliding correlation coefficients between the boreal spring (MAM0) NTA SST index and subsequent boreal summer (JJA0) NTA SST index (measured as the NTA SST persistence). The correlation coefficients between the three curves are shown at the top of the figure. The impact of the previous winter (D–1JF0) ENSO has been removed from the MAM0 NTA SST index using linear regression with respect to the Niño3.4 index, and the horizontal dashed line indicates the 95% confidence level for the sliding correlation coefficients.

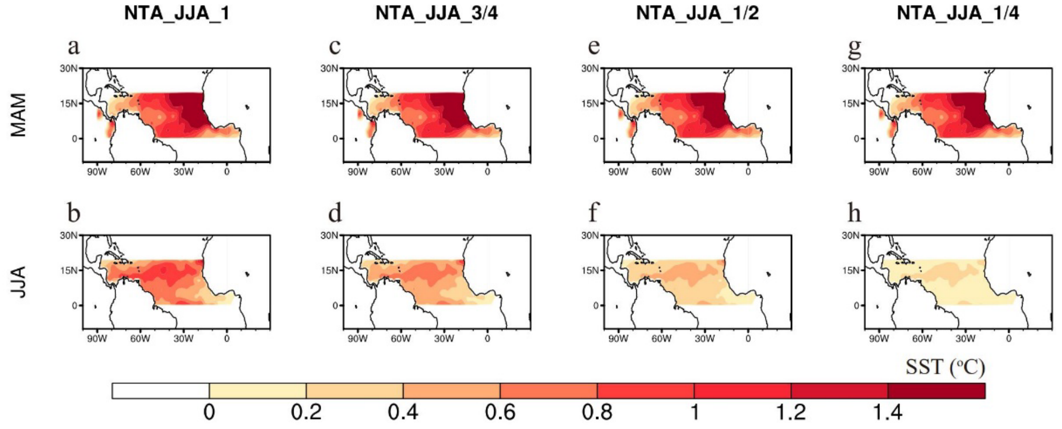

Supplementary Figure 9. Imposed North Tropical Atlantic (NTA) sea surface temperature (SST) forcings in the coupled general circulation model (CGCM) experiments. (**a**,**b**) Spring (**a**) and summer (**b**) NTA SST anomalies, obtained as regressions of spring and summer SST anomalies with respect to the spring NTA SST index for the period 1979–2021, respectively. (**c**,**e**,**g**) Spring NTA SST anomalies as (**a**). In (**d**,**f**,**h**), the magnitude of summer NTA SST anomalies is reduced to (**d**) 3/4, (**f**) 1/2, and (**h**) 1/4 of the magnitude of summer NTA SST anomalies in (**b**).

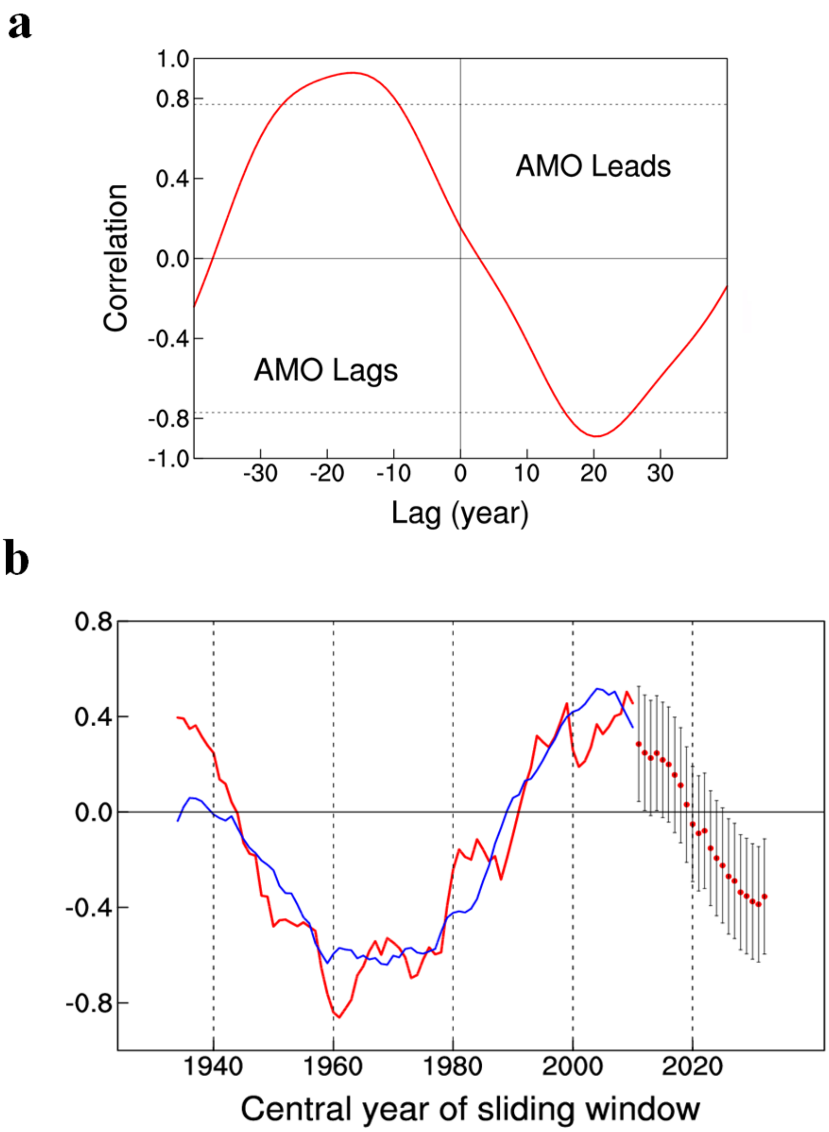

Supplementary Figure 10. Atlantic multidecadal oscillation (AMO)-based linear model for predicting multidecadal North Atlantic Oscillation (NAO) index. (**a**) Lead-lagged correlation between the DJFMAM-averaged NAO and AMO indices (1900–2021). The NAO and AMO indices are smoothed with a 21-year running mean. The dashed lines denote the 95% confidence levels. (**b**) The 21-year running mean of the observed DJFMAM-averaged NAO index (red) from 1932 to 2010, the model fit of the NAO index (blue), and the predicted NAO index in 2011−2031. Vertical bars denote the 95% confidence intervals for the AMO-based linear model. The reason for starting in 1932 is that the original data, which started in 1900, became available from 1911 after a 21-year sliding average. The linear model requires the use of the AMO index 21 years ahead, so the training period of the linear model starts in 1932. Based on the preceding AMO index, we used the linear model to carry out a prediction for multidecadal NAO in 2011–2031.

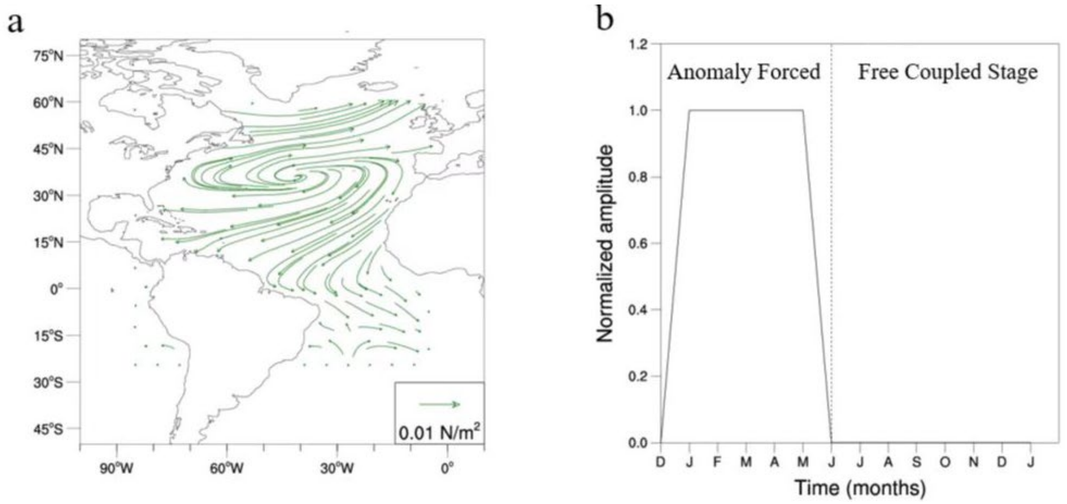

Supplementary Figure 11. Imposed North Atlantic Oscillation (NAO) forcing in coupled general circulation model (CGCM) experiments. (**a**) The spatial pattern of wind stress forcing added to the climatological forcing and applied to each ensemble member in the NAO forcing experiment. The DJFMAM-averaged wind stress forcing is obtained by regressing wind stress anomalies onto the concurrent NAO index. (**b**) Time evolution of the forcing applied during the controlled flux stage (from 1 December(−1) to 31 May(0)) and the fully coupled stage of the NAO forcing experiment (from 1 June(0) to 28 February(1)).
